# CO-DESIGN OF A MOBILE APP FOR CARE PARTNERS WHO MANAGE MEDICATIONS FOR PEOPLE LIVING WITH DEMENTIA

**DOI:** 10.1093/geroni/igae098.0959

**Published:** 2024-12-31

**Authors:** Nicole Werner, Aaron Ganci, Himalaya Patel, Teresa Thuemling, Richard Holden

**Affiliations:** Indiana University, Bloomington, Bloomington, Indiana, United States; Indiana University Indianapolis, Purdue University, Indianapolis, Indiana, United States; Indiana University Bloomington, Bloomington, Indiana, United States; Indiana University Bloomington, Bloomington, Indiana, United States; Indiana University Bloomington, Bloomington, Indiana, United States

## Abstract

For unpaid care partners of community-dwelling people living with Alzheimer’s disease and related dementias (ADRD), medication management is effortful and time-consuming work that often continues over several years. Although this work may be eased by mobile application software (apps), current consumer apps underserve care partners’ needs. Through co-design workshops, our objective was to identify the user requirements for a mobile app supporting at-home medication management for people with ADRD. We conducted five 1-hour virtual co-design workshops with current and recent ADRD care partners. Care partners described their difficulties with medication management, then proposed ideal end-states by hand-drawing storyboards. We co-reflected on care partners’ descriptions and proposals, chose the app’s necessary functions, and digitally sketched corresponding user-facing features. After reviewing the sketches, care partners self-reported attitudes toward the proposed features. We ranked features by desirability, then created a digital user interface prototype. Care partners reviewed the prototype and self-reported adoption intent on a scale from 1 to 5. Seven care partners participated, ages 56-75 (Mdn=63), with 3-14 years’ care-partner experience (Mdn=5.5). Elicited requirements included tracking medication administration, recording behavioral changes, instructing other care partners, and briefing healthcare professionals. The user interface prototype included a medication checklist, observation journal, and contacts management. Care partners self-reported moderately high adoption intent (M=4.2, SD=0.9). By supporting both short- and long-term information needs, the proposed mobile app promotes shared awareness among ADRD care partners and healthcare professionals. This work is foundational to developing the app and assessing usability and utility in situ.

